# A Compact Fiber-Coupled NIR/MIR Laser Absorption Instrument for the Simultaneous Measurement of Gas-Phase Temperature and CO, CO_2_, and H_2_O Concentration

**DOI:** 10.3390/s22031286

**Published:** 2022-02-08

**Authors:** Lin Shi, Torsten Endres, Jay B. Jeffries, Thomas Dreier, Christof Schulz

**Affiliations:** 1 IVG, Institute for Combustion and Gas Dynamics—Reactive Fluids, University of Duisburg-Essen, 47057 Duisburg, Germany; lin.shi@uni-due.de (L.S.); jay@jeffries.org (J.B.J.); thomas.dreier@uni-due.de (T.D.); christof.schulz@uni-due.de (C.S.); 2 High Temperature Gasdynamics Laboratory, Stanford University, Stanford, CA 94305, USA

**Keywords:** combined NIR/MIR laser absorption, laser multiplexing in a mid-IR single-mode fiber, simultaneous multispecies (CO, CO_2_, H_2_O) in situ measurements

## Abstract

A fiber-coupled, compact, remotely operated laser absorption instrument is developed for CO, CO_2_, and H_2_O measurements in reactive flows at the elevated temperatures and pressures expected in gas turbine combustor test rigs with target pressures from 1–25 bar and temperatures of up to 2000 K. The optical engineering for solutions of the significant challenges from the ambient acoustic noise (~120 dB) and ambient test rig temperatures (60 °C) are discussed in detail. The sensor delivers wavelength-multiplexed light in a single optical fiber from a set of solid-state lasers ranging from diodes in the near-infrared (~1300 nm) to quantum cascade lasers in the mid-infrared (~4900 nm). Wavelength-multiplexing systems using a single optical fiber have not previously spanned such a wide range of laser wavelengths. Gas temperature is inferred from the ratio of two water vapor transitions. Here, the design of the sensor, the optical engineering required for simultaneous fiber delivery of a wide range of laser wavelengths on a single optical line-of-sight, the engineering required for sensor survival in the harsh ambient environment, and laboratory testing of sensor performance in the exhaust gas of a flat flame burner are presented.

## 1. Introduction

Laser absorption measurements provide reliable characterization of high-temperature reactive flows such as practical combustion processes [1]. In addition to measurements of the concentration of specific target species, numerous schemes for the determination of temperature, pressure, and gas-phase velocity have been reported, and successful results are well-documented in a variety of review articles [1,2,3,4,5]. The work cited in these reviews shows that the development of solid-state semiconductor diode and quantum-cascade lasers has enabled a wide variety of small portable instruments applicable to numerous practical combustion applications, including a wide range of research and industrial facilities. Use of such instruments in existing industrial facilities and large-scale industrial research test rigs can present significant engineering challenges for the survivability of the sensor in the quest for low-noise laser absorption measurements [1,4].

The purpose of the gas sensor designed here is to enable in situ measurements of the post-combustion gases (CO, CO_2_, H_2_O) as well as temperature in a full-size high-pressure gas turbine burner test rig. The test facility offers a quite harsh environment both inside and outside the combustor. Inside, the combustion chamber is located inside a high-pressure vessel (up to 25 bar) with inlet hot compressed air (up to 770 K) similar to the intake of an aircraft gas turbine combustor can. In the combustion chamber, the temperature is even higher (2000 K) due to the combustion heat release. These conditions challenge sensitive species-selective laser absorption measurements, as the high-temperature distributes the population for any gas species over a very wide range of quantum states and the high-pressure broadens each absorption transition, resulting in a complex spectrum with few (if any) isolated lines. Outside the test facility, the walls of the test rig are quite hot (>150 °C) due to the flames near the combustor walls, which challenges the design of optical access windows and components near the combustor housing. The hot walls of the test rig produce an ambient temperature near 60 °C. Even more challenging is the extreme acoustic noise (up to 120 dB) surrounding the test rig. This acoustic noise exceeds specification of common electronic equipment, produces strong vibrations, and requires remote operation of the equipment. Thus, the thermal and optical engineering solutions for sensitive, species-selective laser absorption measurements that overcome the challenges of the harsh ambient and test environments are an important part of the research reported here.

The design of the sensor reported here builds on a vast library of previous literature. In the past 30 years, there has been an enormous amount of research developing absorption spectroscopy for practical gas-sensing applications [1,2,3,4,5], and the subsequent research literature is exploited to develop the sensor reported here. A complete review of the relevant research is beyond the scope of this introduction, and only a few selected research citations important for the new sensor design are highlighted here.

### 1.1. Selected Background Literature for Absorption Sensing of CO, CO_2_, and H_2_O

The absorption of water vapor in the 2ν_1_ and 2ν_3_ vibrational overtone bands and ν_1_ + ν_3_ combination band overlaps in wavelength with the emission range of mature diode lasers developed in the near-infrared (NIR) for telecommunication. Water vapor, a primary product of hydrocarbon combustion, was a natural target of the early diode laser sensor development for combustion, and there is a rich literature concerning it. More than 20 years ago, Allen’s review [6] highlighted numerous combustion applications of water vapor sensing. In the work reported below, two frequently used and well-studied NIR transitions near 7185.59 cm^−1^ (1391.7 nm) and 6806.03 cm^−1^ (1469.3 nm) are exploited. More recently, Goldenstein et al. demonstrated high-temperature sensitivity using this line pair under high-pressure and high-temperature conditions realized in shock-heated H_2_O/N_2_ mixtures [7], and that work provides the fundamental spectroscopy needed to design the sensor reported here. In addition, the ratio of absorption of water vapor using this pair of laser wavelengths was used to monitor temperature in a rotating detonation engine, illustrating its suitability for harsh environments [8]. The lower-state energy *E*’’ of this pair of transitions differs by 2246 cm^−1^, and thus the ratio of absorption is sensitive to temperature in the 700 to 2400 K range needed for the gas turbine combustion sensing.

CO is an intermediate species, as hydrocarbons are oxidized on the way to becoming the primary product CO_2_. Thus, the CO/CO_2_ ratio is an important indicator for the complete combustion of hydrocarbon fuel. This ratio can increase dramatically if temperature and combustion time is not sufficient for full oxidation in specific operating scenarios of practical devices leading to unwanted toxic emissions of CO. Therefore, measurements of the CO/CO_2_ ratio are invaluable to the designer of new combustor concepts. The second overtone vibrational band of CO (near 1600 nm) also overlaps with the telecommunications wavelength lasers, the first overtone vibrational band (near 2300 nm) overlaps with extended NIR diode lasers, and research on sensors using transitions from both of these bands appear in the literature (e.g., Wagner et al. [9] used the R20 line of CO near 2313 nm to measure spatially resolved CO profiles in atmospheric laminar counter-flow diffusion flame). Unfortunately, these transitions are relatively weak, and the concentration of the intermediate combustion product CO is often much lower than the primary product species CO_2_. Thus, the much stronger transitions in the fundamental vibrational band near 4.6 μm are used here for CO detection. Wavelength-tunable solid-state lasers in the mid-infrared (MIR) with a tuning range that overlaps the CO fundamental band transitions have become available with the development of quantum cascade lasers [10]. CO detection is also complicated by the strong overlap of the fundamental band transitions of CO with those of CO_2_. In the sensor described below, wavelength ranges with different amounts of CO_2_ interference are used to infer the CO absorption.

These laser wavelengths have been used previously to detect CO, e.g., by Spearrin et al. [11], who characterized a QC laser for CO detection in the harsh environment of a pulsed detonation combustor at 4.85 μm (the P(20) transition at 2059.91 cm^−1^) using wavelength-modulation spectroscopy (WMS) with the normalized second-harmonic signal detection (WMS-2f) technique, rendering the measured signal independent of the incoming laser intensity. The encountered gas pressures varying between around 6 and more than 20 bar. With the same technique, the same research group developed a NIR-absorption instrument for the detection of carbon dioxide (CO_2_) with an isolated transition (R(26)) in the ν_1_ + ν_3_ band at 3733.48 cm^−1^ (2.68 μm), with the laser first characterized in shock-tube experiments with pressures between 3 and 12 bar and a large temperature range between 1000 and 2600 K [12]. The instrument was then utilized for CO_2_ detection in a pulse detonation engine (PDE) using ZrO_2_ optical fibers for beam delivery.

Nwaboh et al. [13] used a quantum-cascade laser operated in the intrapulse mode to probe the P(1) line of CO at 2139.4 cm^−1^ (4.67 µm) in gravimetrically prepared gas mixtures to perform an uncertainty analysis for direct absorption spectroscopy-based mole fraction measurements of CO. A reproducibility of 1% and an uncertainty of 4% were demonstrated.

Vanderover et al. [14] used a single MIR QCL covering four selected rovibrational transitions of CO (R(9), R(10), R(17), and R(18) at 2179.77, 2183.22, 2179.24, and 2182.36 cm^−1^, respectively) in the fundamental vibrational band for temperature and concentration measurements from peak absorbance ratios and peak absorbance, respectively. The fractional temperature sensitivity and specific detectivity were demonstrated for gas mixtures prepared in a gas cell (298 K) and a shock tube (1000–3600 K).

Previously, a preliminary version of the sensor used a single quantum-cascade laser (QCL) near 2190.02 cm^−1^ (4566 nm) to detect CO in a full-scale gas turbine burner test rig [15,16]. The CO concentrations varied considerably after ignition, and the laser absorption measurements were in qualitative agreement with the temporal behavior observed by a sampling probe mounted in the free-space exhaust flow just downstream of the optical access port.

### 1.2. Simultaneous Detection of Multiple Species at Atmospheric Pressure

A wavelength-multiplexing scheme that combines multiple lasers onto a single path through the test gases and then separates the lasers by wavelength was exploited very early in the development of laser absorption sensing of combustion gases in the harsh environment of a pulse detonation engine [17]. Diffraction gratings were used to combine multiple lasers onto a common measurement line-of-sight and to demultiplex the wavelengths for individual detection. This approach enables a continuous and simultaneous acquisition of multiple wavelengths. However, this early sensor relied on negligible interference absorption in the ambient air in the optical paths between sensor and engine and between engine and detectors. For the sensor designed here, the light from four lasers (wavelengths spanning from 7185.59 cm^−1^ (1.3917 µm) in the NIR to 2059.91 cm^−1^ (4.8546 µm) in the MIR) is multiplexed onto a common beam path and then coupled into an optical fiber (single mode in the MIR) in a nitrogen-purged enclosure, which allows the light to be delivered to the combustion rig without background absorption of the hot, humid ambient air surrounding the test facility. To our knowledge, wavelength-multiplexing systems using fiber delivery have not previously spanned such a wide range (NIR–MIR) of laser wavelengths (although relevant work is described below using a hollow-core fiber bundle as an alternative solution).

Other lasers are also available in the MIR; for example, using DFB and interband cascade (IC) lasers (not operated simultaneously), Wei et al. [18] were able to measure temperature, CO concentration (via two CO transitions at 2008.53 and 2006.78 cm^−1^, respectively), and CO_2_ concentration (via probing a CO_2_ transition at 2384.19 cm^−1^) in an atmospheric-pressure pilot-stabilized C_2_H_4_/air jet flame. Profiles of these scalars were obtained by translating the sender/receiver units as a whole—and thus the analysis beam—horizontally and vertically through the fixed burner flame.

Nau et al. [19] also used simultaneously a DFB (2.3 µm) and an IC laser (3.1 µm) in a fiber-based absorption spectrometer to measure temperature and concentrations of CO, CH_4_, C_2_H_2_, and H_2_O in an entrained flow gasifier. A wavelength division multiplexing approach using a single ZrF_4_ fiber was used to measure both wavelength regions simultaneously. Weng et al. [20] recently demonstrated simultaneous detection of multiple species (i.e., hydrogen cyanide (HCN), acetylene, and water) by scanning the water vapor spectra using a single NIR laser (1.5 µm). Highly detailed water spectra modelling based on the measured absorption spectra of H_2_O at different temperatures allowed quantification of the small overtone and combinations bands of HCN and acetylene in the same spectral region.

### 1.3. Simultaneous Detection of Multiple Species at Pressures Higher Than Atmospheric

Open literature presentations of solid-state-laser-based simultaneous measurement of multiple species and temperature in the harsh environment of turbulent combustion at high temperature and pressure are scarce. Spearrin et al. [21] recorded two of the major hydrocarbon combustion species, CO and CO_2_ in a scram-jet combustor using both direct absorption and wavelength modulation spectroscopy with a temporal resolution 200 μs (for DA) or 5 ms (for WMS). Through the selection of suitable temperature-sensitive transitions in the MIR at 2059.91 cm^−1^ (4.854 μm) and 2394.6 cm^−1^ (4.176 μm) for CO and CO_2_, respectively, they extended the temperature-sensitive detection range to 800–2400 K in this high-velocity hydrocarbon combustion flow. The laser beams for both CO and CO_2_ detection were coupled into a bifurcated hollow-core optical fiber for transfer to the engine site, where the collimated and collinear beams traversed the optically accessible test section before being split and focused on two separate detectors. Mounting the lasers on vibration-insulated breadboards and the detectors to water-cooled plates and purging all beam paths with N_2_ (including the hollow-core fibers) minimized disturbances from the harsh environment of the flow facility.

In the work of Peng et al. [22], a diode laser absorption instrument was developed combining four diode lasers at NIR and MIR wavelengths for the measurement of water vapor temperature (3920.1 and 4029.5 cm^−1^), CO (P(21) at 2055.4 cm^−1^), and CO_2_ (R(92) at 2394.4 cm^−1^) in a single-ended beam configuration. The beams were directed through the combustion flow channel, while the back-scattered light from the opposite wall was captured by a receiving hollow-core fiber. To enable the beams of all four diode lasers to pass collinearly through the reactive flow, they were fiber-coupled in free space onto a 4-to-1 multimode hollow-core silver-coated glass fiber bundle. 

Cassady et al. [23] developed a very compact and shielded MIR-absorption instrument for the simultaneous measurement of water vapor temperature (via a low- and high-*E*” transition of 3920.06 cm^−1^ (2.551 m) and 4029.59 cm^−1^ (2.482 μm), respectively) and H_2_O, CO (2059.92 cm^−1^ (4.854 µm)), and CO_2_ (2390.52 cm^−1^ (4.175 µm)) concentrations with a high sampling rate of 44,000 samples pre second from the reactive flow in the annular gap of a rotating detonation engine (RDE, operating pressure 2–8 bar) using four co-aligned MIR laser beams and WMS. They were able to perform time-resolved measurements while running the engine at lean to stoichiometric equivalence ratios.

### 1.4. Aim of This Work

The goal of the present work is to develop a laser-absorption sensor for simultaneous CO, CO_2_, and H_2_O measurements in the exhaust of a gas turbine combustor test rig. The instrument is an extension of a single-wavelength transmitter/receiver sensor used for preliminary measurement of CO concentrations in the exhaust flow of a single full-scale gas turbine burner located at the Siemens Energy test center in Ludwigsfelde (Germany) [15,16]. Challenges include the high pressure and temperature inside the combustion zone and the high temperature and acoustic noise in the ambient humid gases near the test rig. The high temperature and pressure in the combustor exhaust produce a complex absorption spectrum with a paucity of isolated lines, where most transitions are strongly overlapped by pressure broadening. The overlap of the fundamental absorption bands of CO and CO_2_ exacerbates this problem. The acoustic noise (up to 120 dB) exceeds the operating limits of typical electronic devices, and the sensor must be packaged to protect electronics and optics. The high ambient temperature (up to 60 °C) also exceeds typical electronics and optical equipment specifications, and the sensor package is thus assembled on water-cooled breadboards. The packaged sensor must be small enough for easy transport with installation by two operators with only minor adjustment for optical path alignment at the test site.

While our previous work [15,16] focused on the challenging measurement environment associated with gas turbine combustor testing and solved issues such as optical access and thermal and acoustic isolation of the measurement equipment, the focus in this work is on enhancing the measurement capabilities of the sensor. In the present work, a combined NIR/MIR laser-based absorption spectrometer is described for the simultaneous measurement of line-of-sight-averaged gas-phase CO, CO_2_, and H_2_O in combustion exhaust, and temperature is inferred from the ratio of two H_2_O absorption transitions. The sender unit has a small footprint due to separately placing electronics and optical parts on two stacked temperature-controlled boards and mounting inside a N_2_-purged box to avoid background water vapor absorption. The instrument uses two MIR lasers near 2059.91 cm^−1^ (4.8546 μm, centered around the P(20) CO transition) and 2190.02 cm^−1^ (4.5562 μm, centered around the R(12) CO transition), previously explored by Spearrin et al. [11]. Simultaneously, two NIR diode lasers probe two water vapor transitions (7185.59 and 6806.03 cm^−1^) with differing temperature sensitivities previously used by Goldenstein et al. [7,8]. The beam delivery can be either free-space or via optical fibers compatible with the various wavelengths emitted by the installed laser sources. The receiver optics and detectors are also mounted in a metal box purged with an expanding flow of dry nitrogen for keeping the interior cool and free of water vapor.

### 1.5. Organization of This Paper

This manuscript is organized as follows: After a short introduction of the spectroscopic basics of laser absorption spectroscopy, a discussion of some specific details such as line selection, and the used fitting approach, a detailed outline is given of the experimental setup, including sending, auxiliary, receiving, free-beam guiding unit, and burner. Finally, the initial demonstration of the setup is described by presenting measurements on a standard McKenna burner to validate temperature and species-concentration values (CO, CO_2_, and H_2_O) for literature-known operating conditions.

## 2. Theoretical Background

### 2.1. Absorption Measurements in High-Temperature and -Pressure Environments

High temperature spreads the population of the target species into a large ensemble of quantum states, and this diluted population means less absorption for any specific optical transition. At the same time, high pressure broadens optical transitions and blends the individual transitions so that an absorption measurement with a narrowband laser often includes contributions from multiple transitions. Thus, the high-temperature, high-pressure measurement conditions require understanding of the temperature- and pressure-dependent absorption spectrum of the target species and the potential interference from other components of the gas along the optical path. For the sensor design and data analysis, we rely on modeling of the absorption spectrum using the HITRAN/HITEMP database [24,25] augmented with prior laboratory work on the specific laser wavelengths used for detection (see more details and citations in the Introduction section and below).

### 2.2. Basics of Laser Absorption

The theory of direct absorption spectroscopy is well-understood and is only briefly reviewed to clarify the notation and units [26,27]. For a more detailed introduction, we refer to textbooks on the subject [28,29]. Measurements are based on the Beer–Lambert law, which describes the relation between incident laser light intensity $I_{0}$ and transmitted laser light intensity $I_{t}$ at wavenumber $\tilde{\nu }$ as it passes through a gas medium on a path length L as:

(1) $$\left(\frac{I_{t}}{I_{0}}\right)=e^{-\alpha_{\tilde{\nu }}}=e^{-k_{\tilde{\nu }}L},$$

where the spectral absorbance $\alpha_{\tilde{v}}$ is the product of L and the spectral absorption coefficient $k_{\tilde{v}}$ (cm^−1^), which is defined according to:

(2) $$k_{\tilde{\nu }}=p\;x_{i}\;S\left(T,{\tilde{v}}_{0}\right)\;\phi_{\tilde{\nu }}$$

where p (bar) is the pressure, $x_{i}$ the mole fraction of the absorbing species i, $S\left(T,{\tilde{v}}_{0}\right)$ (cm^−2^ bar^−1^) the line strength dependent on temperature T and line-center wavenumber ${\tilde{\upsilon }}_{0}$ (cm^−1^), and $\phi_{\tilde{\upsilon }}$ (cm) the line-shape function, which is normalized with $\int_{-\infty }^{+\infty }\phi_{\tilde{\upsilon }}d\tilde{\nu }\equiv 1$.

For a single transition, the absorbance can be integrated as:

(3) $$A_{i}=\int_{-\infty }^{+\infty }\alpha_{\tilde{\nu }}\;d\tilde{\nu }=p\;x_{i}\;S_{i}\left(T\right)\;L.$$

The line strength is given by:

(4) $$S_{i}\left(T\right)=S_{i}\left(T_{0}\right)\frac{Q\left(T_{0}\right)}{Q\left(T\right)}\left(\frac{T_{0}}{T}\right)\exp{\left(-\frac{hcE_{i}^{\prime \prime }}{k}\left(\frac{1}{T}-\frac{1}{T_{0}}\right)\right)}^{}\frac{1-\exp{\left(\frac{-hc{\tilde{\nu }}_{0,i}}{kT}\right)}^{}}{1-\exp{\left(\frac{-hc{\tilde{\nu }}_{0,i}}{kT_{0}}\right)}^{}}\;,$$

where T is an arbitrary temperature, $T_{0}$ is a reference temperature, h is the Planck constant (J s), c is the speed of light (cm/s), k is the Boltzmann constant (J/K), $E_{i}^{\prime \prime }$ is the lower-state energy (cm^−1^), and $Q\left(T\right)$ is the partition function, which is also temperature-dependent and can be approximated with the following polynomial:

(5) $$Q\left(T\right)=a+bT+cT^{2}+dT^{3}.$$

Coefficients (a,b,c,d) for CO, CO_2_, and H_2_O from Ref. [30] are used. 

The less congested absorption spectrum of water vapor (as compared to CO_2_) is used to infer temperature from the ratio of two absorption transitions with different ground state internal energy. Along a common optical path, the water mole fraction and pressure are the same for both transitions; thus, the ratio of the two integrated absorbance values can be simplified to the ratio of the respective line strengths:

(6) $$R=\frac{A_{1}}{A_{2}}=\frac{S_{1}\left(T\right)}{S_{2}\left(T\right)}=\frac{S_{1}\left(T_{0}\right)}{S_{2}\left(T_{0}\right)}\exp{\left(-\frac{hc}{k}\left(E_{1}^{\prime \prime }-E_{2}^{\prime \prime }\right)\left(\frac{1}{T}-\frac{1}{T_{0}}\right)\right)}^{}\frac{1-\exp{\left(\frac{-hc{\tilde{\nu }}_{0,1}}{kT}\right)}^{}}{1-\exp{\left(\frac{-hc{\tilde{\nu }}_{0,1}}{kT_{0}}\right)}^{}}\frac{1-\exp{\left(\frac{-hc{\tilde{\nu }}_{0,2}}{kT_{0}}\right)}^{}}{1-\exp{\left(\frac{-hc{\tilde{\nu }}_{0,2}}{kT}\right)}^{}}.$$

If the two selected transitions have similar wavelengths, the ratio of two integrated absorbances can be approximated while maintaining high accuracy as follows:

(7) $$R=\frac{S_{1}\left(T_{0}\right)}{S_{2}\left(T_{0}\right)}\exp{\left(-\frac{hc}{k}\left(E_{1}^{\prime \prime }-E_{2}^{\prime \prime }\right)\left(\frac{1}{T}-\frac{1}{T_{0}}\right)\right)}^{}.$$

To infer the highest temperature measurement accuracy, the value should be as large as possible over the expected temperature range.

(8) $$\left|\frac{\frac{dR}{R}}{\frac{dT}{T}}\right|=\left(\frac{hc}{k}\right)\frac{\left|E_{1}^{\prime \prime }-E_{2}^{\prime \prime }\right|}{T}$$

From Equation (7), the temperature can be obtained by:

(9) $$T=\frac{\frac{hc}{k}\left(E_{2}^{\prime \prime }-E_{1}^{\prime \prime }\right)}{\frac{hc}{kT_{0}}\left(E_{2}^{\prime \prime }-E_{1}^{\prime \prime }\right)+\ln\frac{S_{2}\left(T_{0}\right)}{S_{1}\left(T_{0}\right)}+\ln R}.$$

In Equation (2), the line-shape function $\phi_{\tilde{\nu }}$ (cm) is a convolution of Doppler and collisional broadening [31]:

(10) $$\phi \left(\tilde{\nu }\right)=\phi_{D}\left({\tilde{\nu }}_{0}\right)\frac{a}{\pi }\int_{-\infty }^{+\infty }\frac{e^{-y^{2}}}{a^{2}+{\left(w-y\right)}^{2}}dy=\phi_{D}\left({\tilde{\nu }}_{0}\right)V\left(a,w\right),$$

where $V\left(a,w\right)$ is the Voigt function that can be numerically approximated [32]. The Voigt parameter as a measure for the relative significance of Doppler and collisional broadening is defined as w indicates the non-dimensional line position $\phi_{D}\left({\tilde{\nu }}_{0}\right)$ is the Doppler line center magnitude at ${\tilde{\nu }}_{0}$ and y is an integration variable:

(11) $$a=\frac{\sqrt{\ln2}\Delta {\tilde{\nu }}_{c}}{\Delta {\tilde{\nu }}_{D}}.$$

(12) $$w=\frac{2\sqrt{\ln2}\left(\tilde{\nu }-{\tilde{\nu }}_{0}\right)}{\Delta {\tilde{\nu }}_{D}},$$

(13) $$\phi_{D}\left({\tilde{\nu }}_{0}\right)=\frac{2}{\Delta {\tilde{\nu }}_{D}}\sqrt{\frac{\ln2}{\pi }},$$

(14) $$y=\frac{2u\sqrt{\ln2}}{\Delta {\tilde{\nu }}_{D}}$$

The collision-broadened line width $\Delta {\tilde{\nu }}_{c}$ depends on the pressure and the product of the sum of the mole fraction for each collision partner species B and its collisional broadening coefficient $2\gamma_{B}$:

(15) $$\Delta {\tilde{\nu }}_{c}=P\underset{B}{\sum }x_{B}2\gamma_{B},$$

which varies with temperature

(16) $$2\gamma_{B}\left(T\right)=2\gamma_{B}\left(T_{0}\right){\left(\frac{T_{0}}{T}\right)}^{N},$$

where $T_{0}$ is the reference temperature and N  is the temperature coefficient. The temperature-dependent Doppler broadening is defined by:

(17) $$\Delta {\tilde{\nu }}_{D}={\tilde{\nu }}_{0}\left(7.1623\times 10^{-7}\right){\left(\frac{T}{M}\right)}^{0.5},$$

where M is the molecular mass in g/mol.

When temperature has been determined by the two-color ratio method and pressure and optical path length are known, the species mole fractions can be determined from the absorption of a single transition of known spectroscopic parameters. In cases, where no isolated transition can be measured, the concentration of the target species is inferred from model calculations matching the measured absorption spectrum.

## 3. Wavelength Selection and Data Analysis

An important part of wavelength-multiplexed sensor design is the selection of laser wavelengths to target sections of the absorption spectrum of the target gas. In the ideal case, each laser is chosen to scan in wavelength across an individual transition of the target species that is free of interference from other chemical components of the gas along the laser line-of-sight. As noted before, in high-pressure, high-temperature hydrocarbon combustion gases, such isolated absorption transitions are not found for CO, CO_2_, and H_2_O detection. For water vapor, the pressure-broadened target transitions are blended with other water vapor transitions. For CO and CO_2_, the situation is more complicated, as the fundamental absorption bands of these two species strongly overlap, and the CO concentration is typically much lower than CO_2_ in combustion product gases. For all three species, determination of the zero-absorption transmitted laser intensity is not straightforward. Center wavenumbers and wavelengths, line strengths, and lower-state energies of the four target transitions utilized are listed in Table 1. The two NIR water vapor transitions have been previously used by several authors, e.g., [1,7,8,33], and the two MIR transitions have been used for CO and CO_2_ by the Hanson group [27].

**Figure 1:**
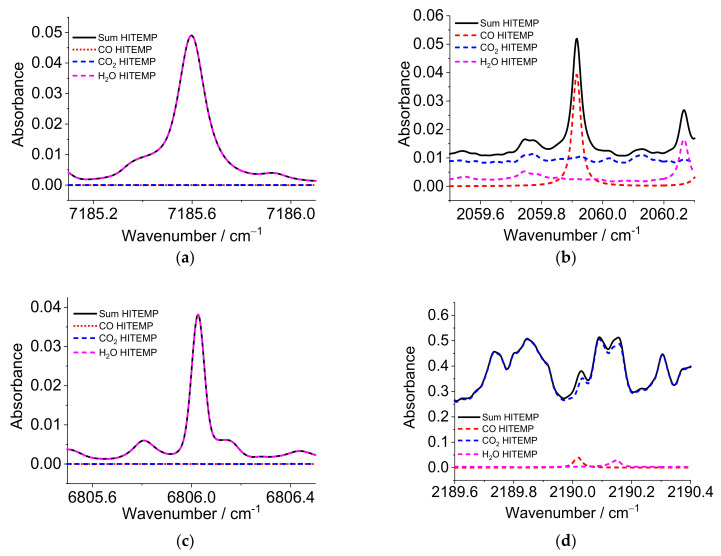
Simulated absorbance spectra for each of the four wavelength ranges used at 1.01 bar, 2000 K, 0.1% CO, 8% CO_2_, 18% H_2_O mole fraction with an optical path length of 60 mm for the spectral regions near 7185.59, 6806.03, 2059.91, and 2190.02 cm^−1^, respectively. The NIR spectra (**a**,**c**) consist of several water absorption lines, and each spectrum is dominated by a single strong transition. The MIR spectrum (**b**) is dominated by an isolated CO transition that builds on a CO_2_ and water background. MIR spectrum (**d**) consists mainly of several CO_2_ absorption lines, with some overlap of isolated CO and water transitions. The large number of CO_2_ absorption lines in this region and their superposition results in a significant baseline for the CO measurement.

Using the HITEMP database [25], Figure 1 shows spectra simulations in the region of the selected wavelengths at 1 bar with a temperature of 2000 K and an optical pathlength of 60 mm. The assumed mole fractions of CO, CO_2_, and H_2_O are 0.1, 8, and 18%, respectively, typical for the burned gas effluent from hydrocarbon combustion. Figure 1a,c show the region around the two target water lines used for thermometry. The water vapor lines near 1391.67 nm (7185.59 cm^−1^) and 1469.29 nm (6806.03 cm^−1^) are free of significant interfering absorption from other components of the gas flow such as CO and CO_2_. However, several water transitions overlap within the depicted scan range. Most importantly, note the absorbance never goes to zero even at pressures as low as 1 bar, making the determination of the zero-absorption baseline an important task. The absorbance shown in the scan range of Figure 1b is primarily CO absorption with only modest contributions from CO_2_, while the scan range in Figure 1d is the reverse. Thus, simultaneous fitting of the two MIR scan ranges can return the CO and CO_2_ concentrations, especially since the gas temperature is known from the ratio of the two water vapor measurements.

**Figure 2:**
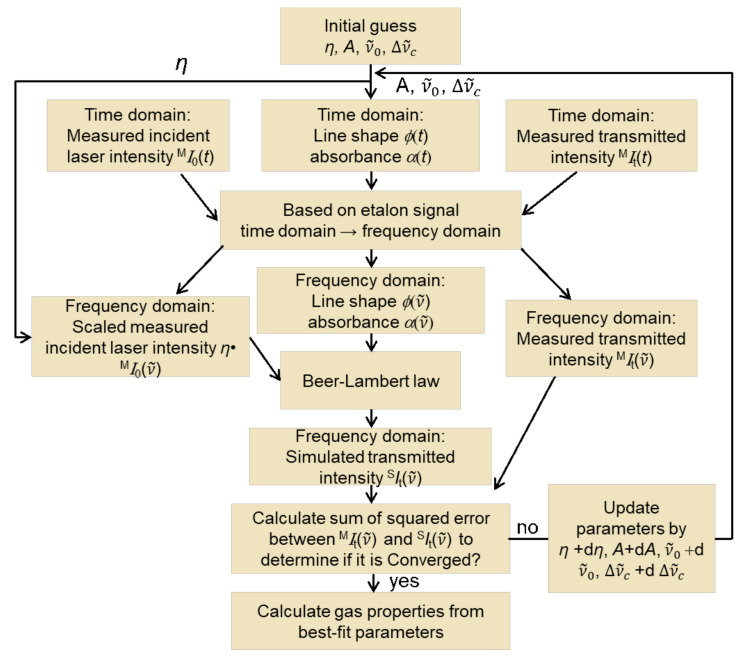
Algorithm for iterative spectra fitting by comparing simulation and measurement of the transmitted intensity versus time (i.e., wavelength) of a wavelength-scanned laser.

The zero-absorption baseline is determined for the sensor by first measuring the laser transmission over the scanned-wavelength region, with nitrogen purging of the measurement line-of-sight before and after the measurement. However, detection efficiency and laser intensity transmitted for the benign no-combustion measurement can vary from the combustion gas measurement by differences in optical alignment and potential beam steering in the hot combustion gases (especially in the target gas turbine combustor test rig). Therefore, we assume a linear loss term *η* (baseline scaling factor) for each laser intensity, which is determined by iterative spectral fitting over the scan range using the algorithm described in Figure 2.

The purpose of the algorithm in Figure 2 is the calculation of the transmitted laser intensity. The algorithm determines the integrated absorbance A for each line, the line-shape function $\phi \left(\tilde{\nu }\right)$, and the baseline scaling factor η. Initial guesses are needed. For the integrated absorbance A (Equation 3) for each individual transition, line-center wavenumber ${\tilde{\nu }}_{0}$, and the collisional broadening coefficient $\Delta {\tilde{\nu }}_{c}$ values from the HITEMP database [25] are used. The relationship between laser scanning time and frequency is characterized by using an etalon placed in a third beam path (cf. Figure 4), so that the line-shape function, absorbance, and the measured incident and transmitted laser intensity can be converted from the time domain to the frequency domain. The simulated transmitted intensity versus frequency ^S^*I*_t_$\left(\tilde{\nu }\right)$ is obtained with the Beer-Lambert law and baseline measured without combustion ^M^*I*_0_$\left(\tilde{\nu }\right);\;$ to account for non-absorption losses with combustion, this baseline intensity is scaled by a fit parameter, *η*. After comparison of the simulated and the measured transmitted intensities versus frequency ^S^*I*_t_$\left(\tilde{\nu }\right)$ and ^M^*I*_t_$\left(\tilde{\nu }\right)$, the gas properties are determined from best-fit parameters.

## 4. Experiment

### 4.1. Layout of the NIR/MIR Four-Color Laser Absorption Instrument

**Figure 3:**
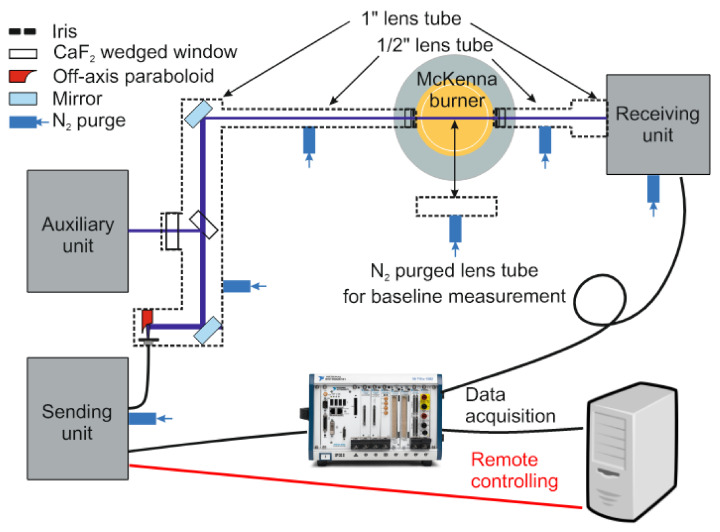
Optical layout of MIR/NIR laser absorption instrument.

Figure 3 shows the schematic of the instrument consisting of the sending, auxiliary, receiving, and free-beam guiding units. The sending, receiving, and free-beam guiding units are enclosed in acrylic glass or metal boxes and purged with dry nitrogen to protect all optics from dust deposition and avoid spurious absorption from water vapor and CO_2_ outside the probe region. Indium trifluoride (InF_3_) single-mode MIR-transmitting optical fibers (Thorlabs, P3-32F-FC-1) are used to deliver the laser light from the sending unit to the free-beam guiding unit composed of optical windows and purged lens tube components with a varying diameter (Thorlabs lens tubes, 1” and 1/2” diameters). The attenuation of the InF_3_ fiber in the MIR range is <0.35 dB/m and <0.52 dB/m in the NIR range. However, it should be noted that fiber for NIR radiation does not behave as a single-mode fiber, which causes limitations in the ability to focus the beam and the quality of the beam profile, but these effects do not cause any crucial limitation for the application here.

The laser beams leaving the sending unit (see below) are collimated by an off-axis paraboloid (Thorlabs, RC02APC-P01, Bergkirchen, Germany) mounted on an *xy* translation stage fixed at the free-beam-guiding lens tube. Inside the lens tube, the light beams are steered with a gold-coated mirror and split into two parts by an optically polished CaF_2_ wedge (3 degrees, Korth Kristalle GmbH, Altenholz, Germany). The reflected part is transmitted through a wedged window (1°) towards the auxiliary unit. The transmitted laser beams are guided via 1/2”-diameter lens tubes through the flame and then towards the receiver unit. Each lens tube is closed with wedged (0.5°) CaF_2_ windows (Thorlabs, WW50530) and apertures (Thorlabs, SM05D5D) and flushed with dry nitrogen to avoid absorption by room air water vapor. The flat flame is operated using a McKenna burner (Holthuis & Associates, Sebastopol, CA, USA) mounted on a lab jack (Thorlabs, L490/M) for height adjustment. 

All detector outputs in the sending and receiving units are acquired by two DAQ cards (PXIe-6124, National Instruments, Austin, TX, USA) in the PXI system, which also worked as a function generator for the current modulation of the lasers. The system also controls and remotely operates stepper motors for mechanical translation stages.

### 4.2. Sending Unit

**Figure 4:**
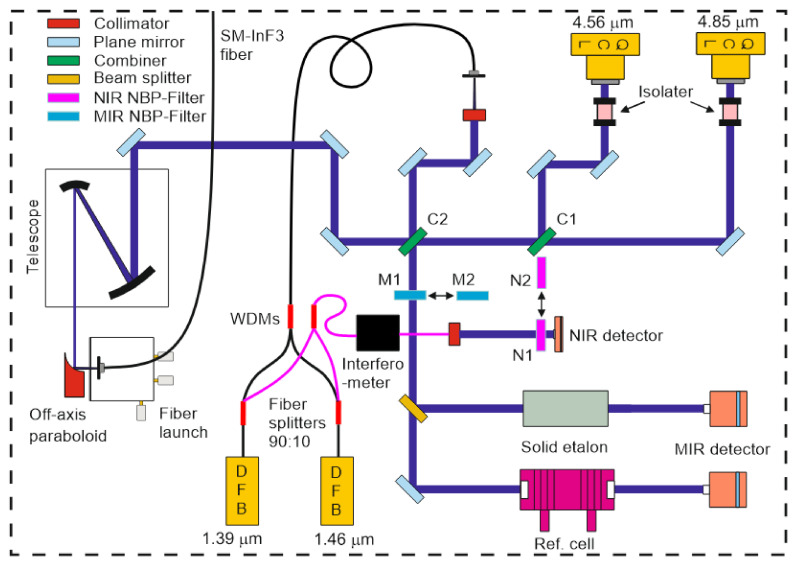
Optical layout of the sending unit.

Figure 4 shows a more detailed sketch of the optical layout inside the sending unit. The cw-QCL (Alpes Laser, 40 mW) with emission wavelength around 4.85 µm is housed together with a collimator in a sealed laser mount. The laser is fixed on a home-made water-cooled heat sink. A second cw-QCL (Hamamatsu L12004-2190H-C, 20 mW, Herrsching, Germany) with an emission wavelength centered around 4.56 µm is packaged in a sealed laser mount on a water-cooled heat sink (Hamamatsu A11709-02). An aspheric ZnSe lens (Hamamatsu, A11331-02) inside a precision zoom unit (Thorlabs, SM1ZM) can also be translated perpendicular to the laser beam path (*z* direction) with an *xy* translation mount (Thorlabs, ST1XY-D). The QCLs are thermoelectrically cooled with Peltier elements. To attain stable performance, the heat sinks are connected in series and water cooled with a chiller (Thermo Fisher Scientific, 200 W, Waltham, MA, USA). Two free-space adjustable narrowband optical isolators (Thorlabs, I4500W4 and I4730W5) are used to shield the lasers from back reflections, thus reducing intensity noise and mode hopping. The free beams from both lasers are collinearly combined on a narrowband filter (C1, Spectrogon, NB5040-155 nm, Täby, Sweden) that is used as a long-pass beam combiner transmitting the 4.85 µm beam and reflecting the 4.56 µm beam. In comparison with a 50:50 beam splitter, this filter achieves better overall optical efficiency of 75% for each beam. However, the filter may cause ghosting and beam distortion due to etaloning and limited surface optical quality, respectively.

For the water transitions, two polarization-maintaining (PM) fiber-coupled DFB (distributed-feedback) lasers with emission wavelengths of 1.392 and 1.469 µm (NEL, 20 mW) are clamped into butterfly laser mounts (Arroyo, 203) and thermoelectrically cooled with Peltier elements. For a compact arrangement, they are stacked onto a platform mounted above other optical elements. To prevent damage of long sections of optical fibers, they are coiled into storage reels (Thorlabs, FSR1) separately fixed on the cover. With two 1 × 2 PM fiberoptic couplers (Thorlabs, PN1310R2A1 and PN1480R2A1), the two beams at 1.39 and 1.47 µm are separately split into two PM fractions of 90% and 10% each. To monitor the mode quality and to obtain frequency markers to convert scan time to relative wavelength, the 10% beams are combined using a single-mode (SM) fiber wavelength-division multiplexer (Thorlabs, WD202C-APC, WP9850A) and together propagate through a SM fiber-coupled interferometer (Micron Optics, FFP-I, 0.8 GHz). Its output is collimated and free-space recorded by an amplified InGaAs photodetector (Thorlabs, 0.8 mm^2^ active area, max. 11 MHz bandwidth). In front of the detector, two narrow bandpass filters (Thorlabs, FB1400-12 as N1, FB1480-12 as N2 in Figure 4) are placed in a dual-position filter holder slider with resonant piezoelectric motors (Thorlabs, ELL6K) such that either 1.39 or 1.47 µm can be selected for etalon analysis to determine the wavelength tuning characteristics. The motor of the filter slider is remotely controlled during the measurement.

The 90% beams are combined using another PM wavelength-division multiplexer (Thorlabs, WP9850A). The coupled beams with the two NIR wavelengths are collimated with an aspherical lens (Thorlabs, C280TMD-C) in a fiber-launch system (Thorlabs, KT110/M). They are subsequently combined with the two collinear propagating MIR beams by a second bandpass filter (Spectrogon, BP-4700-600 nm) operated as combiner C2, with most of the MIR beams being transmitted and the NIR beams reflected off the front surface. For wavelength and tuning characterization, a fraction of the incoming free-space MIR beams is reflected towards a solid germanium etalon and a reference absorption cell, respectively. The detectors record each laser beam through respective narrow bandpass filters (Laser Components, SNB-4860-001793 as M1 and Spectrogon, NB4560-135 nm as M2 in Figure 4) installed in another dual-position filter slider equipped with a resonant piezoelectric motor (Thorlabs, ELL6K). The filtered beams are split into two parts: One passes through the low-pressure reference cell to determine the spectral position of the narrow CO absorption line during a wavelength scan whose peak is identified with the absolute wavelength listed in the HITEMP database. The other part is guided to the 7.62-cm-long solid Ge etalon (free spectral range (FSR) 0.016 cm^−1^). The known fringe spacing enables conversion of tuning time into relative laser wavelength. Information from both channels can be combined to convert scan time into absolute wavelength.

All collinearly combined NIR and MIR beams are then steered to a parabolic mirror (Thorlabs) and are focused into a SM MIR InF_3_ patch fiber (Thorlabs, P3-32F-FC-1). Due to its small numerical aperture of 0.26, a six-fold reflective beam telescope (Thorlabs, BE06R/M) reduced the beam diameter to approximately 100 µm. This telescope significantly reduced the coupling losses in the fiber. By precisely adjusting the positions of the collimators in front of the PM fiber outputs of the NIR lasers (and the QCL at 4.56 µm), the difference in beam divergence of the four collimated beams can be slightly minimized to achieve better focus quality and reduce coupling losses. 

**Figure 5:**
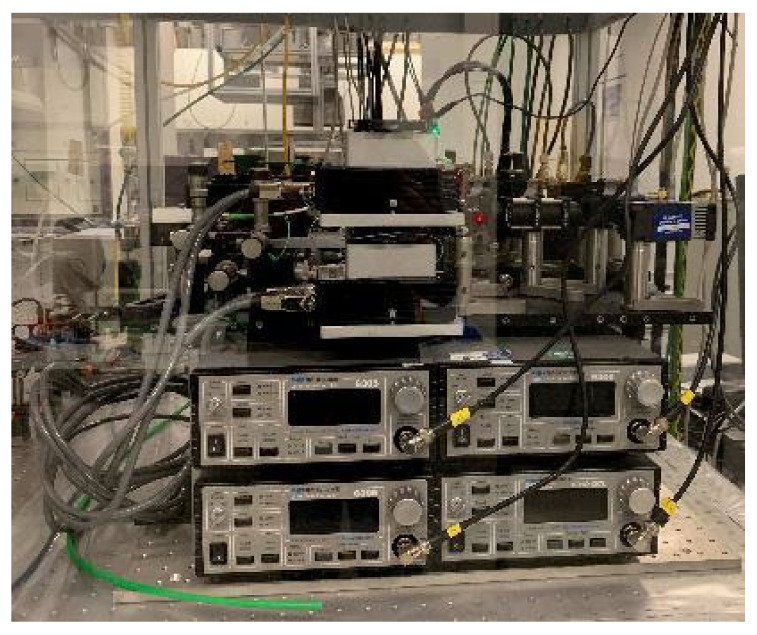
Side view of the sending unit mounted on an optical breadboard with the laser controllers below.

All four lasers are driven by individual controllers (Arroyo, 6300 series) including temperature stabilization and injection current control. The optical setup is installed on a water-cooled aluminum optical board (450 × 600 mm^2^, Thorlabs), which rested on four aluminum profiles with passive vibration isolators (Figure 5). Below this plate, two fans provided air circulation within the unit. The remaining space accommodated controllers and power supplies for lasers and detectors. 

### 4.3. Receiving Unit

**Figure 6:**
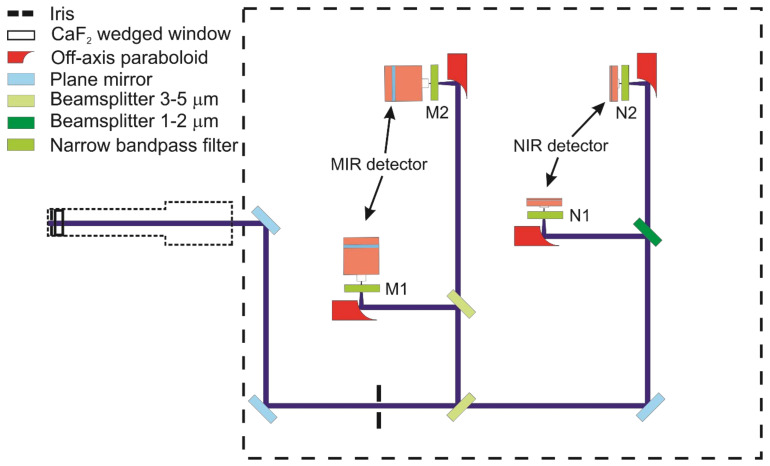
Schematics of the receiving unit.

The receiving unit is illustrated in Figure 6. An aperture (Thorlabs, SM05D5D) and a CaF_2_ wedged window (Thorlabs, WW50530) are fixed inside of a 12.7 mm diameter adjustable lens tube to allow the laser beam to couple directly into the N_2_-purged environment after passing the burner. Thus, the absorption path is confined within the flame gases and minimizes interference of ambient water vapor in the beam path outside of the flame. Optics and detectors of the receiving unit are contained inside an aluminum box (10-mm wall thickness) with a 25.4 mm diameter optical access port originally designed for shielding the probe from excessive acoustic noise and thermal load when placed close to the gas turbine test site. Lens tubes and the whole receiver box are purged with dry nitrogen supplied through a Vortex tube (Vortec, 208-25H, Cincinnati, OH, USA) that simultaneously keeps the internal atmosphere cooled to approx. 293 K even at elevated environmental temperature.

The four incoming collinearly propagating transmitted laser beams are split by a 45:55 pellicle beam splitter (Thorlabs, BP145B4), originally designed for the 3–5 µm wavelength range, which also has a transmittance for the NIR-lasers of 85% and 63% near 1392 and 1469 nm, respectively. In the MIR detection beam path, a second 3–5 µm 45:55 pellicle beam splitter delivers a reflected and transmitted beam towards two TE-cooled IR photovoltaic detectors (Vigo Systems, 2 mm diameter active area, 1 MHz bandwidth), where they are focused with parabolic mirrors (Thorlabs, MPD019-M01). Two narrow bandpass filters (Spectrogon, NB4560-135 nm as M1 and Laser Components, SNB-4860-001793 as M2 in Figure 6) transmit the respective beams and block unwanted background emission from hitting the detectors. The fraction of the NIR beams transmitted through the first pellicle beam splitter to the NIR detection side is again divided into two separate beams with a third 45:55 pellicle beam splitter for 1–2 µm (Thorlabs, BP145B3) and directed to two InGaAs free-space amplified photodetectors (Thorlabs, PDA20CS2, 3.14 mm^2^ active area, max. 11 MHz bandwidth). Narrow-bandpass spectral filters (Thorlabs, FB1480-12 as N1 and FB1400-12 as N_2_ in Figure 6) are mounted in front of the respective detectors. All the optics are fixed on a threaded aluminum breadboard (300 × 300 mm^2^) inside the closed box. 

### 4.4. Auxiliary Measurements

**Figure 7:**
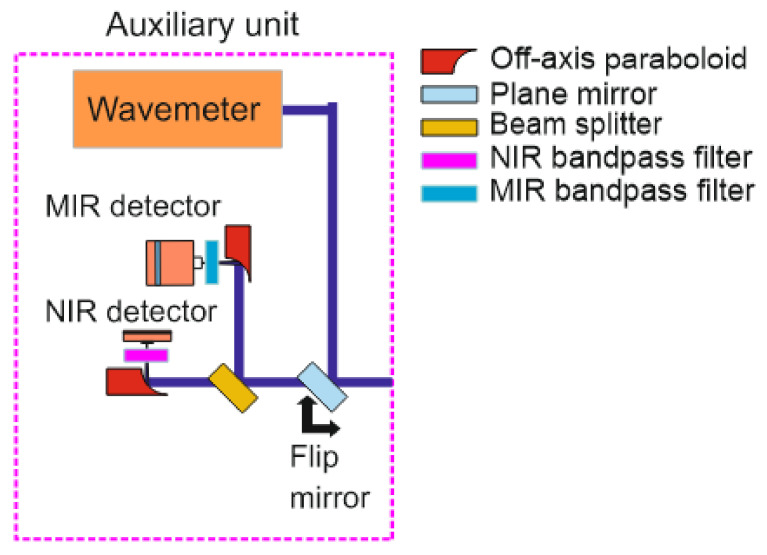
Schematics of the auxiliary unit.

A split-off fraction of the incoming beams enters the auxiliary unit (details Figure 7) through a separate side window in the lens tube of the sending unit, where they are again split by a 45:55 pellicle beam splitter (coated for 3–5 µm; Thorlabs, BP145B4). Depending on the wavelength, they are then focused with parabolic mirrors (Thorlabs, MPD00M9-M01, *f* = 15 mm) on an InGaAs free-space amplified photodetector (Thorlabs, PDA20CS2, 0.8 mm^2^ active area, max. 11 MHz bandwidth) or a TE-cooled IR photovoltaic detector (Vigo Systems, 1 mm active diameter, 1 MHz bandwidth), respectively. A flip mirror in front of the beam splitter can in addition switch the optical path towards a MIR wavemeter (Bristol 621B) for monitoring the absolute wavelength.

### 4.5. Burner Facility

Flat methane/air premixed flames are stabilized on a water-cooled McKenna burner at atmospheric pressure. It is equipped with a central sintered bronze matrix of 60 mm diameter for delivering the fuel/air mixture and an outer 6 mm-wide ring-shaped matrix for providing a coannular flow of dry nitrogen. All gases are metered by calibrated mass flow controllers (Bronkhorst, Kamen, Germany), while the cooling water flow for the burner matrix is circulated at 16 °C by a thermostat (Huber, Minichiller 600 OLÉ, Offenburg, Germany). For vertical translation with respect to the spatially fixed optical path for the absorption measurements, the burner is mounted on a lab jack. The height above burner is measured with a caliper, whose two arms are fixed to the lab jack and the breadboard, respectively, with a zero value when the traversing analysis beam is just halfway clipped by the burner surface. Premixing of fuel and oxidizer well upstream of the burner inlet improved the flatness of the flame, as is visible from flame chemiluminescence. 

## 5. Results

To validate the performance of the wavelength-multiplexed NIR/MIR absorption sensor, measurements are conducted in the burned gases above the flat flame at conditions identical to measurements presented by Weigand et al. [34], where gas-phase temperatures were obtained by coherent anti-Stokes Raman scattering (CARS) of molecular nitrogen, and CO, CO_2_, and H_2_O concentrations are calculated assuming local thermodynamic equilibrium; three stoichiometric and slightly fuel-rich flames labeled No. 17, 18, 19, and 20 ae studied and provide validation data for measurements with the sensor developed here. Table 2 lists flow rates (in standard liters per minute, slm) of CH_4_, air, and co-flow N_2_ and the corresponding equivalence ratio *ϕ* for the premixed CH_4_/air flames. Also listed are the adiabatic flame temperatures (*T*_ad_) and the measured N_2_ CARS temperatures, with the latter varying in the range 1883–2100 K. The three flames have equilibrium concentrations of CO ranging from 0.31 to 4.3%, CO_2_ varying between 6.5 and 9.1%, and water vapor at approximately 19% (Table 2).

The measurement line-of-sight is parallel to the burner surface and crosses the center of the burner at a height of 15 mm (matching the height above the burner for the CARS measurements). At this height, temperature gradients in the exhaust above the flame front are negligible [35]. The four lasers are tuned at 10 Hz with individual inverse sawtooth current ramps to cover each of the respective absorption line shapes from which integrated line areas are calculated. The signal voltages from each detector are acquired at 10 MHz and averaged for 30 s.

**Figure 8:**
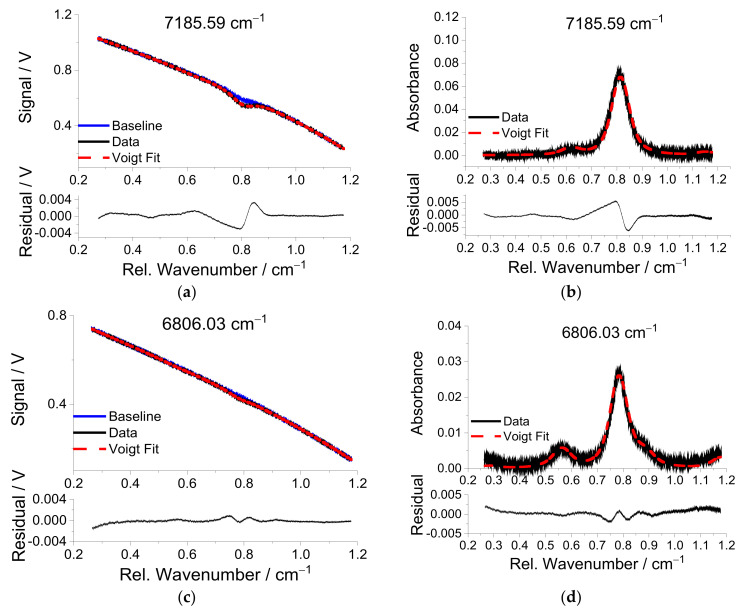
Water vapor transmitted signal (black lines in (**a**,**c**)) and calculated absorbance (black lines in (**b**,**d**)) over relative wavenumber at the H_2_O center wavelengths 7185.59 and 6806.03 cm^−1^, respectively, for the premixed flame no. 17 (Table 2). The separately measured baseline (blue lines in (**a**,**c**)) and fitted Voigt functions (dashed red lines) are also shown. The residuals shown in the lower section of each figure are between original data and fitted line shapes.

Temperatures are measured with two-line thermometry using water vapor transitions with center wavenumber positions 7185.59 and 6806.03 cm^−1^ from Table 1. Figure 8 shows the transmitted signal (upper row) and the corresponding absorbance (lower row) over the relative wavenumber for these two lines for flame no. 17 in Table 2. As can be seen from Figure 8a,c, the “corrected” baseline that is scaled by measured reference baseline without absorption matches very well in the far wings of the lines due to the large tuning range of the NIR lasers (>1 cm^−1^) and the fact that the transitions are isolated from interference of neighboring lines from other species. The absorption line at 7185.59 cm^−1^ has significant absorption at room temperature from ambient water vapor. Although the sender unit is also covered by an acrylic glass box on the optical table, the observation of a small absorbance indicates imperfect purging of the sensor unit. In contrast, the high energy of the lower state of the transition near 6806.03 cm^−1^ reduces the sensitivity to interference from ambient water vapor, and its baseline scan is free of the corresponding interference absorbance (Figure 8c). As can be seen from Figure 8b,d, the fitted absorbance line shape agrees with the measured one fairly well, with a maximum residual of 0.5% of the peak absorbance.

For CO detection in the MIR, interference by CO_2_ and water vapor present a challenge. Since the water absorption lines are not significantly disturbed by other interfering species and temperature is measured using two-line thermometry, any water vapor interference can be calculated from the corresponding absorbance of one of the water transitions.

**Figure 9:**
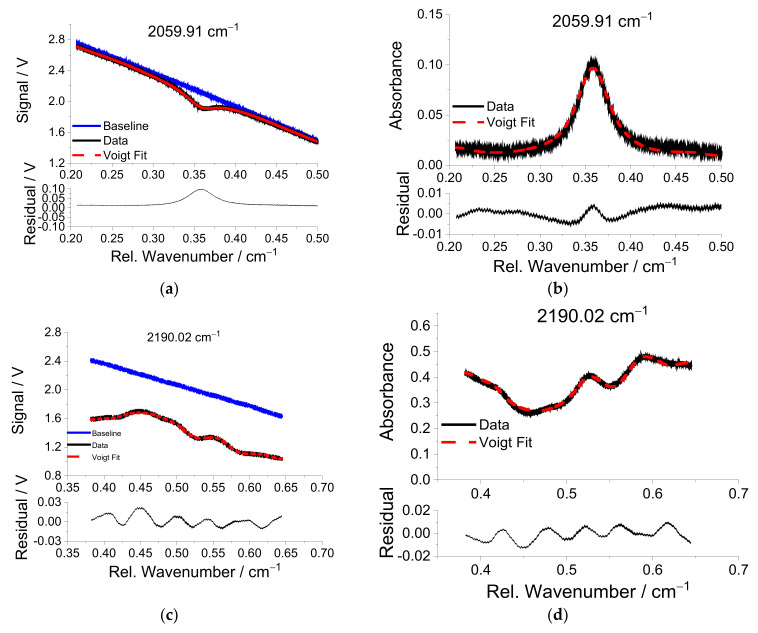
Transmitted signal and calculated absorbance originating from CO, CO_2_, and H_2_O over the relative wavenumber at the CO center wavelengths 2059.91 cm^−1^ (**a**,**b**) and 2190.02 cm^−1^ (**c**,**d**) for the premixed flame no. 17.

For the MIR scan of the CO lines, the interference of CO_2_ must be considered. Using the temperature determined from the water vapor, the CO and CO_2_ concentrations are simultaneously fit to the two MIR scanned-wavelength absorbances. The CO line near 2059.91 cm^−1^ experiences only slight interference from CO_2_, while the spectral region around the CO line near 2190.02 cm^−1^ at elevated temperatures is dominated by CO_2_ absorbance. Figure 9b and d show that the Voigt fits match the measurements well, with maximum residuals below 2% of the absorbance. Although there is a significant residual in the baseline in Figure 9d, the best-fit spectral profile matches well with the measured data suggesting a fairly complete CO_2_ spectroscopic model extracted from the HITEMP data base in this spectral range. However, the residual between measurement and modeled spectrum still is not “flat”, signifying discrepancies in CO_2_ spectral line positions and relative intensities in the data base.

**Figure 10:**
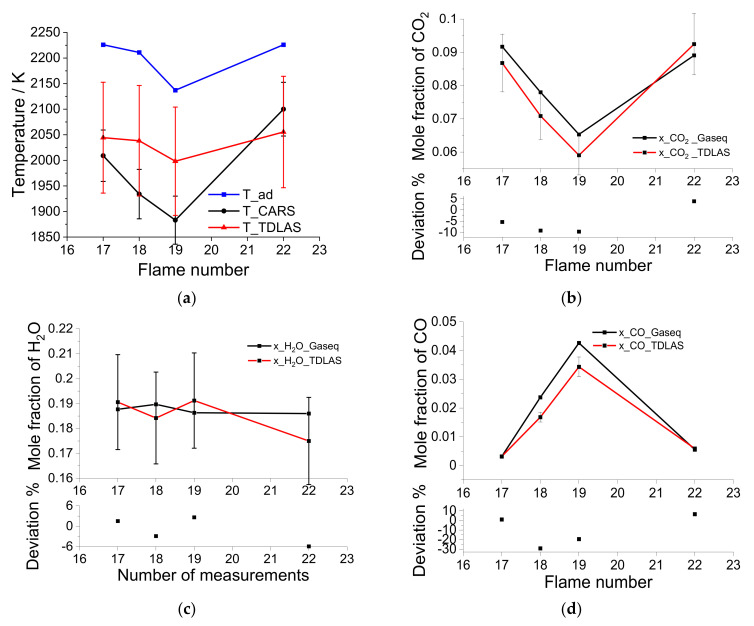
Measured temperatures (**a**) and concentration of CO_2_ (**b**), H_2_O (**c**) and CO (**d**) in comparison to the temperatures measured by CARS and the simulated equilibrium concentrations from Ref. [34]. The measurement uncertainties for the CARS measurements were assumed to be 2.5% according to Ref. [34]. The measurement uncertainty for temperature measurements using two-line absorption thermometry with 1.4-µm lasers is discussed in the relevant literature as being between 2.8% [7] and 6.7% [36]. In Ref. [29], the temperature deviation of identical burners is estimated at 2.5% as an additional source of error. Therefore, and as no detailed accuracy analysis was performed as part of this study, we estimated the error for our temperature measurements to be ~5%. Mole fraction measurements using laser absorption methods under best conditions (only CO_2_ in N_2_ at room temperature) have been reported in the literature with accuracies up to 4% [37]. In Ref. [36], an accuracy of 10% was achieved for the determination of water vapor mole fraction in the exhaust gas of a flat Mckenna-type burner. In Ref. [7], an accuracy of 4.7% for the determination of shock-tube-heated water vapor was reported. Since the conditions and the used setup in Ref. [36] best match ours, we estimated the error for our mole fraction measurements to be ~10%.

Table 2 and Figure 10 compare the measurements of the laser absorption sensor with the CARS temperatures and equilibrium concentrations for CO, CO_2_, and H_2_O [34]. Laser absorption recovers line-of-sight averaged temperature and concentration values, while the CARS technique provides spatially resolved measurements (within a roughly cylindrical probe volume of a few millimeter in length and a diameter of approx. 500 µm). Laser absorption temperature measurements are higher than the reported CARS measurements for lower flow rates and lower than CARS temperature for the high-flow-rate flame (No. 22). These statements lose their significance when the measurement uncertainties (plotted in Figure 10) are considered; note the error bars of CARS and the absorption measurements (for details, see figure caption) strongly overlap. Nerveless, it can be concluded that the temperature can be successfully measured using the sensor within the measurement uncertainties.

Laser absorption measurements of the concentrations of CO, CO_2_, and H_2_O shown in Figure 10b–d agree well with calculated equilibrium values from Gaseq [35]. For only the two rich operating points (flame #18 and #19), strong deviations between the equilibrium calculations and the CO concentration results are observed, which are larger than can be explained by the measurement uncertainty. In this case, there are apparently unconsidered systematic errors. Since the dynamic range of the CO signal is very large, small nonlinearities in the detector could cause these systematic errors at higher concentrations. Another explanation could be inaccuracies in the spectroscopic line parameters for the selected CO transitions or deficiencies in the determination of the CO concentration via the equilibrium calculation using GasEq. Since these deviations occur at concentrations of CO that are irrelevant for the operation of gas turbines (only a few ppm CO may be generated) and the chosen laser was designed for this low-CO range, this deviation does not represent a limitation for the applicability of the sensor.

## 6. Summary

The design and demonstration of a compact, modular fiber-coupled laser absorption instrument for simultaneous remote measurement of temperature, concentrations of water vapor, carbon dioxide, and carbon monoxide is reported. The instrument is designed for remote operation in test facilities with extremely harsh ambient conditions. The assembly of the sender and receiver units on water-cooled breadboards allows them to be enclosed with a purged atmosphere to protect against the high-temperature humid gases and the extreme acoustic noise of the gas turbine combustion test facility. The gas-phase temperature is determined by two-line thermometry using two well-known NIR transitions near 7185.59 cm^−1^ (1.3917 µm) and 6806.03 cm^−1^ (1469.3 nm) without spectral interference from other primary product species; the H_2_O concentration is then inferred from the total absorbance of either transition. The concentrations of CO and CO_2_ are detected using two MIR laser wavelengths near 2059.91 cm^−1^ (4.8546 µm) and 2190.02 cm^−1^ (4.5562 µm). These two spectral regions have quite different amounts of CO_2_ interference, enabling simultaneous modeling of the absorbance measured by both MIR lasers using the temperature determined by water vapor absorbance to infer the CO and CO_2_ concentrations.

The two NIR and two MIR lasers are multiplexed into an optical fiber (single-mode in the MIR) for delivery from the sending unit to the measurement line-of-sight. The four laser beams are combined into an indium trifluoride (InF_3_) single-mode optical fiber, delivering all lasers colinearly overlapped along the absorption path length. We believe the efficient, low-noise laser-multiplexing into the InF_3_ fiber is unique to this sensor.

An algorithm is developed for finding the baseline for wavelength-scanned direct absorption spectroscopy; even when within the scan range of each laser, no true zero-absorption baseline can be recovered.

The sensor operation is validated by measurements in the burned gases in the exhaust of a premixed flat flame previously studied by CARS [34]. The best-fit laser absorption measurements of gas temperature and CO, CO_2_, and H_2_O concentration agree well with the CARS measurements and equilibrium calculated concentrations. The validation experiment illustrates that the combined NIR/MIR laser absorption sensor is suitable and ready for field applications.

## Figures and Tables

**Table 1:** Spectroscopic parameters of chosen main transitions.

Line	Species	${\tilde{\nu }}_{0}/{\mathrm{cm}}^{-1}$	*λ*/nm	*S* (296 K)/(cm^−2^/bar)	*E*″/cm^−1^	Transition
1	H_2_O	7185.59	1391.67	1.93 × 10^−2^	1045.59	Q(6)
2	H_2_O	6806.03	1469.29	6.32 × 10^−7^	3291.2	P(17)
3	CO	2059.91	4854.58	8.65 × 10^−1^	806.38	P(20)
4	CO	2190.02	4556.17	7.04	299.77	R(12)

**Table 2:** Operating conditions of the four premixed CH_4_/air flames from Weigand et al. [34] examined in this work. Adiabatic flame temperatures and species mole fractions from equilibrium calculations (EQ) as well as measured CARS temperatures as listed from [34]. CARS temperatures were measured at 15 mm HAB. Measured laser absorption temperature and species mole fractions from this work are listed below the reference values.

Flame No.	CH_4_/slm	Air/slm	Co-flow N_2_/slm	*ϕ*	*T*_ad_/K	*T*/K CARS Laser Abs	*x*(H_2_O) EQ Laser Abs	*x*(CO_2_) EQ Laser Abs	*x*(CO) EQ Laser Abs
17	2.55	24.14	10.55	1.0	2226	2009 2226	0.1877 0.1906	0.0917 0.0868	0.0031 0.0031
18	2.55	22.00	9.7	1.1	2211	1934 2211	0.1897 0.1842	0.0780 0.0709	0.0237 0.0169
19	2.55	20.20	8.99	1.2	2137	1883 2137	0.1863 0.1912	0.0653 0.0590	0.0426 0.0343
22	3.42	32.4	14.15	1.0	2226	2100 2226	0.1860 0.1750	0.0891 0.0925	0.0055 0.0059

## Data Availability

Not applicable.
